# Supplementary material for: Maternal immune activation imprints translational dysregulation and differential MAP2 phosphorylation in descendant neural stem cells
Source: Mol Psychiatry. 2025 Feb 3;30(7):2994–3007. doi: 10.1038/s41380-025-02905-5 (PMC12185331; doi:10.1038/s41380-025-02905-5)
Supplement: Supplementary file 1 — Supplementary Information [file 41380_2025_2905_MOESM1_ESM.docx]

**Maternal Immune Activation imprints translational dysregulation and differential MAP2 phosphorylation in descendant neural stem cells**

Sandra M. Martín-Guerrero^4*^, Ph.D., María Martín-Estebané^1*^, Ph.D., Antonio Lara-Ordóñez^1^, Ph.D., Miguel Cánovas^1^, M.S., David Martín-Oliva^2^, Ph.D., Javier González-Maeso^3^, Ph.D., Pedro R. Cutillas, Ph.D ^¶4^., Juan F. López-Giménez, Ph.D.^¶1^

^1^Instituto de Parasitología y Biomedicina “López-Neyra” (IPBLN-CSIC). E-18016 Granada. Spain.

^2^Departamento de Biología Celular, Facultad de Ciencias, Universidad de Granada, Granada. Spain.

^3^Department of Physiology and Biophysics, Virginia Commonwealth University School of Medicine, Richmond, VA 23298, USA.

^4^Centre for Cancer Genomics and Computational Biology, Barts Cancer Institute, Queen Mary University of London, Charterhouse Square, London EC1M 6BQ, UK.

^¶^To whom correspondence may be addressed:

Juan F. Lopez-Gimenez Pedro R. Cutillas

IPBLN-CSIC Centre for Haemato-Oncology

Av. del Conocimiento 17 Barts Cancer Institute

E-18016, Granada Queen Mary University of London

Spain Charterhouse Square, London EC1M 6BQ

Tel. 00-34-958181657 United Kingdom

jf.lopez.gimenez@csic.es Tel. 00-44-2078825555

[p.cutillas@qmul.ac.uk](mailto:p.cutillas@qmul.ac.uk)

**^*^**these authors contributed equally to this work.

**Running title: MIA imprinting in descendant neural stem cells**

**SUPPLEMENTARY INFORMATION (SI)**

**Supplementary Figure 1. Poly I:C treatment protocol to induce maternal immune activation in pregnant mice.**

**Supplementary Figure 2. Pluripotency markers in NSCs.**

**Supplementary Figure 3. Time-lapse differentiation process of NSCs cell lines. Supplementary Figure 4. Synaptogenesis analysis using rabies virus monosynaptic tracing methodology.**

**Supplementary Figure 5. Differences in transcriptomics data between Saline and Poly I:C groups using non-adjusted p-value.**

**Supplementary Figure 6. Significant identifications in omics data (transcriptomic, proteomic and phosphoproteomic data).**

**Supplementary Figure 7. Protein levels of glia and neuron markers in the proteomic data set.**


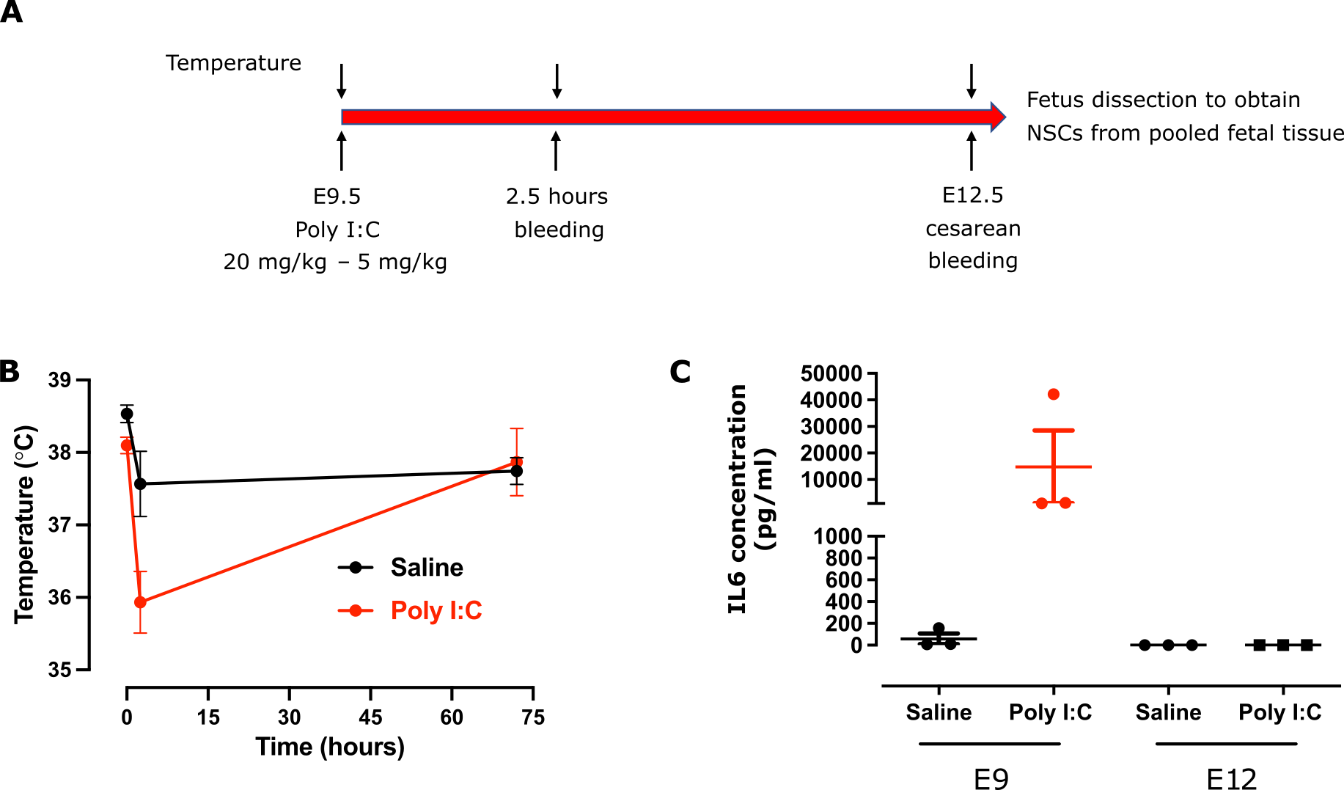


**Supplementary Figure 1. Poly I:C treatment protocol to induce maternal immune activation in pregnant mice. A.** Timeline of the protocol from the moment of the Poly I:C acute administration to the cesarean surgery to access fetal tissue samples. Top arrowheads indicate the time points when the animal temperature was measured. Bottom arrowheads indicate the time points when blood samples were collected for subsequent IL-6 determinations.

**B.** Variation in pregnant mice temperature throughout the protocol depicted in **A**. Each point represents the mean ± SEM of 3 animals. **C.** IL-6 concentration found in the serum from blood extracted from animals at the time points indicated in **A**. Each point corresponds to an individual animal, and error bars represent the SEM.


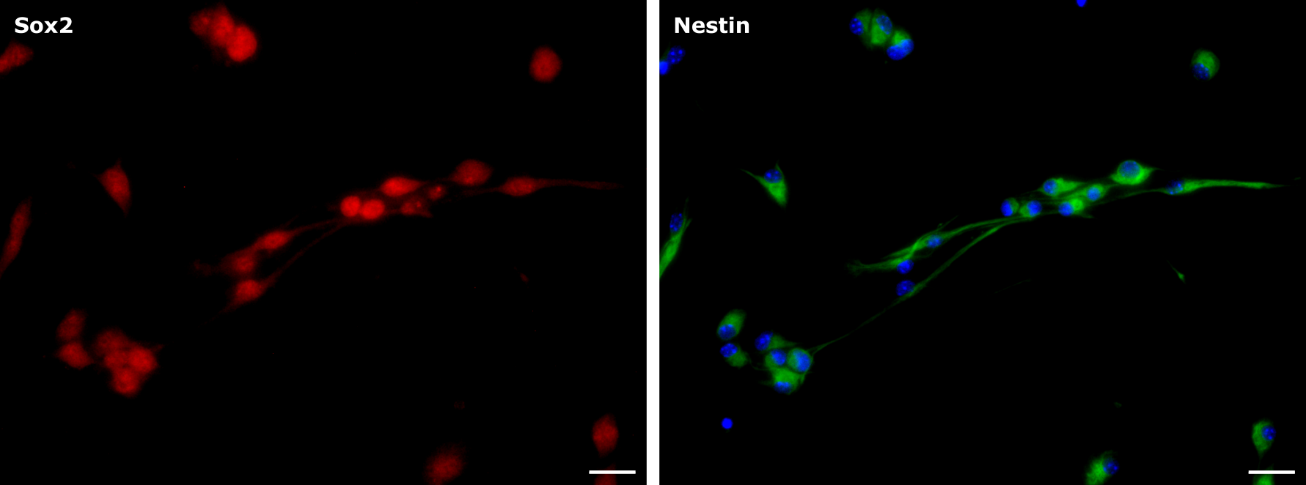


**Supplementary Figure 2. Pluripotency markers in NSCs.**

Representative image of NSC lines obtained from fetuses probed against pluripotency markers Sox 2(red) and Nestin (green). Cell nuclei are labelled with Hoescht 33342 (blue). Scale bar: 25 µm.


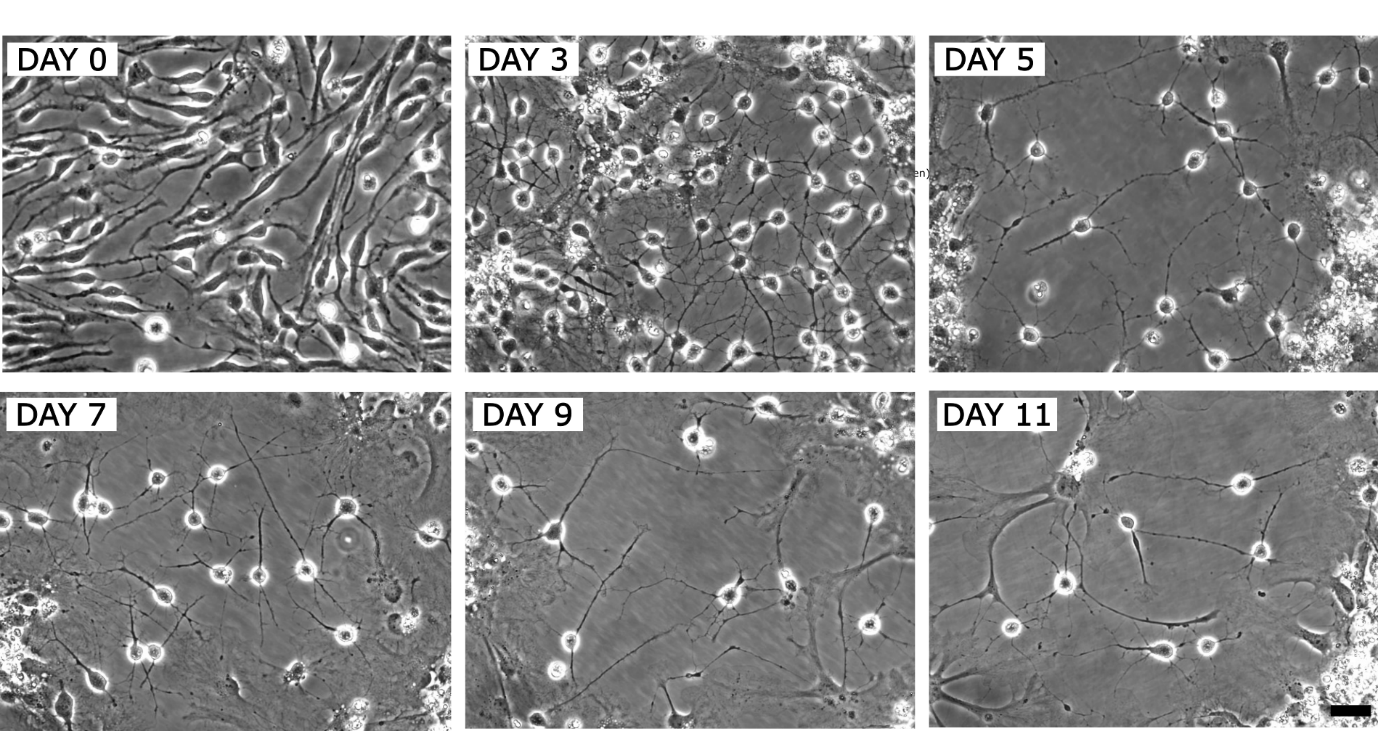


**Supplementary Figure 3. Time-lapse differentiation process of NSCs cell lines.** Representative images of different time points (days of culture) during the differentiation process of NSCs. Scale bar: 50 µm.


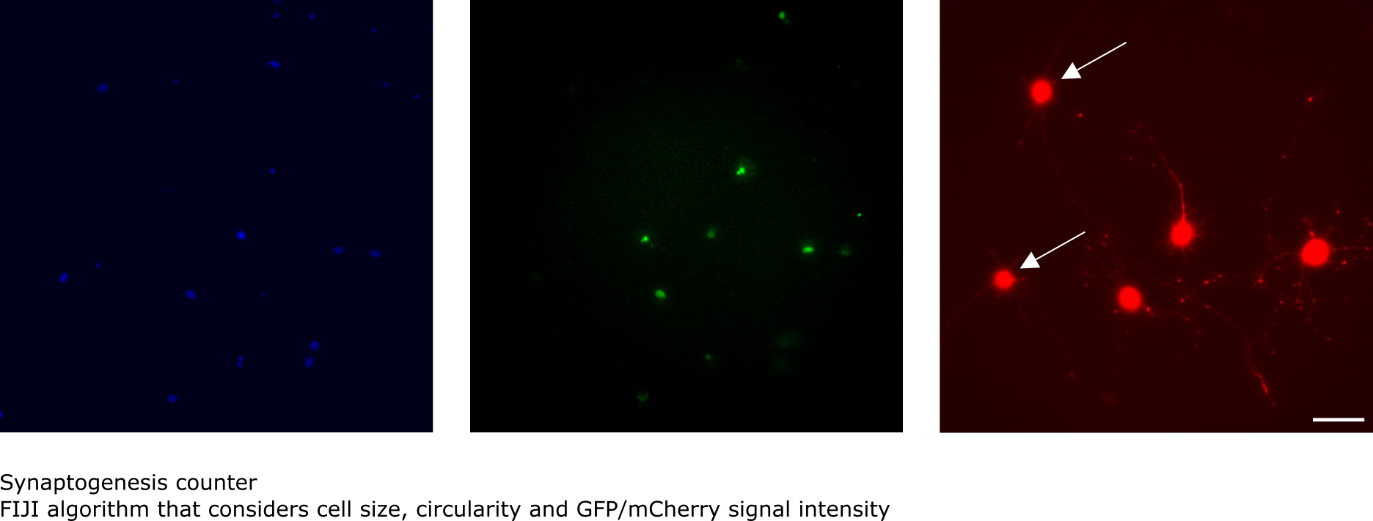


**Supplementary Figure 4. Synaptogenesis analysis using rabies virus monosynaptic tracing methodology.**

Representative microscopy fields are indicated by arrowheads, highlighting neurons that do not co-express green (TVA receptor) and red (recombinant rabies virus) signals. On differentiation day 3, cells were infected with lentivirus to induce the expression of the TVA receptor, along with GFP and rabies capsid glycoprotein (shown in green). Subsequently, on day 5, cells were infected with the recombinant rabies virus expressing mCherry protein (shown in red). Finally, on day 7, image acquisition from living cells was conducted after staining the cell nuclei with Hoechst 33342 (shown in blue). The nuclei staining visualizes the total population of cells within the microscopy field. Among the cells expressing the mCherry protein (red), there were cells displaying both GFP and mCherry fluorescent signals, as well as cells emitting only the mCherry signal. The image corresponding to the red channel, on the right panel, is intentionally overexposed in this case to show the dendritic arborization and thus the neuronal nature of these cells. Scale bar: 50 µm.


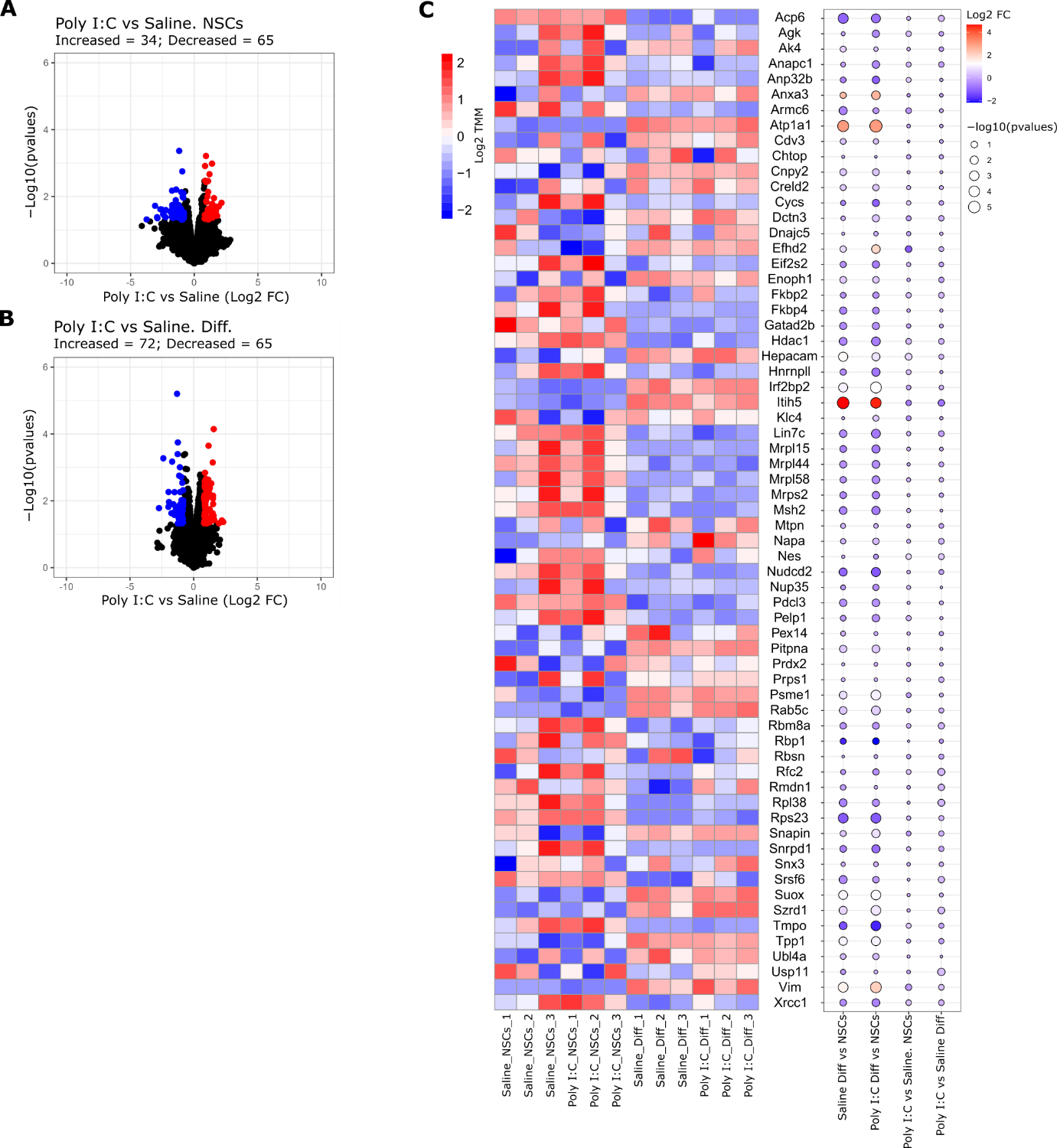


**Supplementary Figure 5. Differences in transcriptomics data between Saline and Poly I:C groups using non-adjusted p-value.** Differences comparing Poly I:C and Saline before and after differentiation of NSCs. **A, B.** Volcano plots showing transcriptomics data from NSCs and differentiated cells. X-axis shows relative change expressed as Log2 Fold Change (FC) of Poly I:C versus Saline NSCs or differentiated cells. Y-axis show statistical significance expressed as -Log10 pval (non-adjusted p-value). Red and blue dots correspond to genes that changed significantly respect to Saline group (pval <0.05 and Log2 FC<-0.8 or Log2 FC> 0.8, respectively); black dots represent genes or proteins that did not match with the filtering criteria (pval > 0.05, Log2 FC > −0.8 or Log2 FC <0.8). **C**. Heatmap showing the normalized counts (expressed as Log2 TMM) in Saline and Poly I:C NSCs before and after the differentitation progress. The genes indicated in the heatmap belong to the significant proteins identified in Fig3B (bottom volcano plot) and Fig3C. Dot plot indicates the Log2 Fold change in the comparison indicated and the statistical significance (pval, non-adjusted p-value). The size of the dots indicated the level of significance for each comparison, indicated as -Log10 pvalues (pval). The colour of the dots indicates the relative change expressed as Log2 FC each comparison.

**
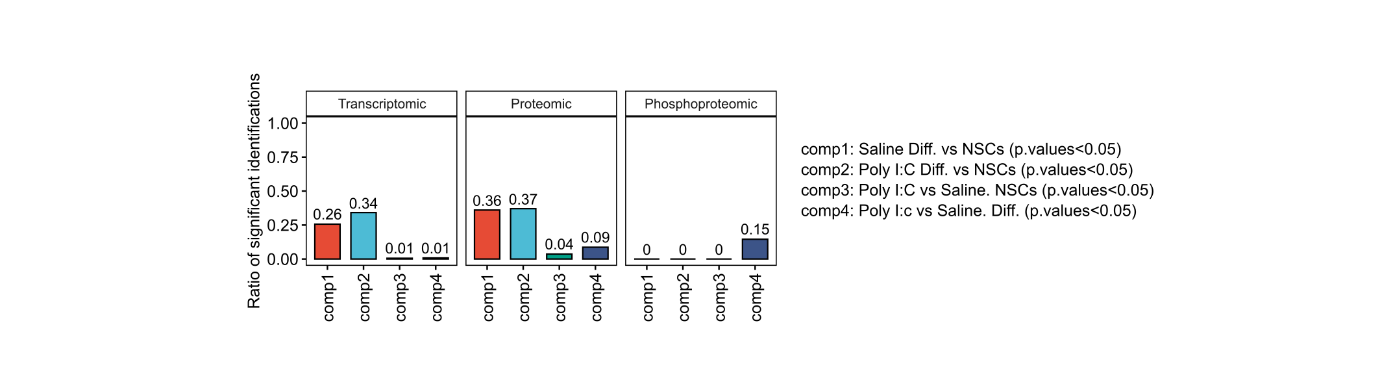
**

**Supplementary Figure 6. Significant identifications in omics data (transcriptomic, proteomic and phosphoproteomic data).** Ratio of significant identifications (transcripts, proteins or phosphopeptides) to total identifications in all the omics performed for this study. Only identifications with a p-values<0.05 and an absolute fold change of 0.8 were considered. For phosphoproteomic analysis, only Poly I:C and Saline differentiated cells were processed for LC-MS/MS analysis as indicated in the main manuscript (comp4).


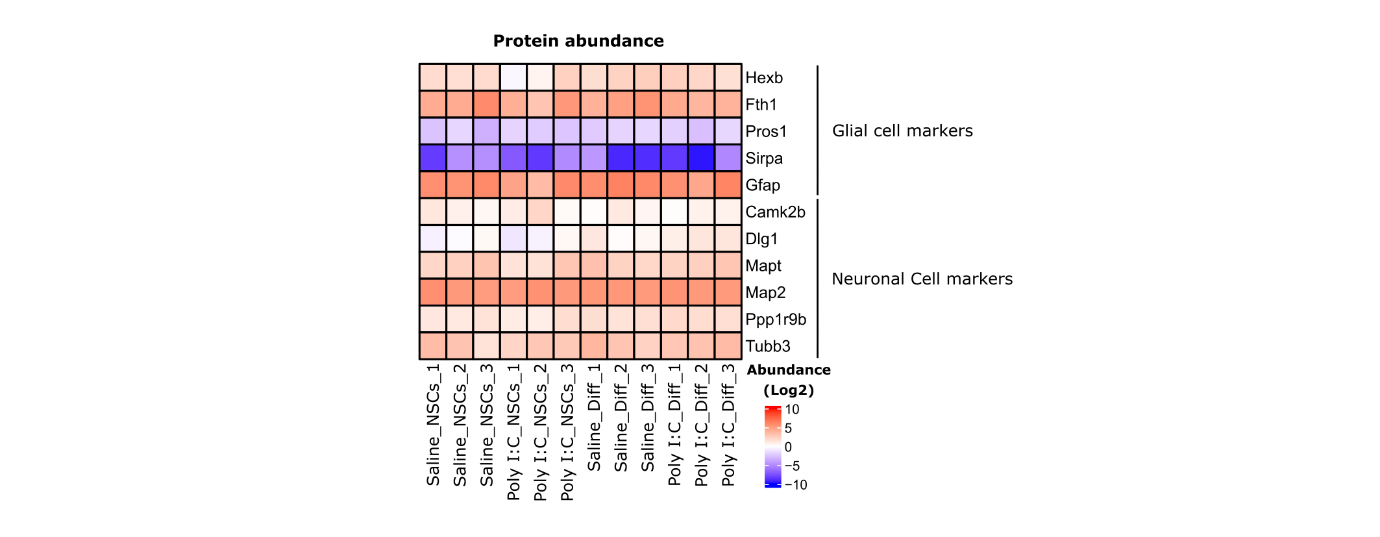


**Supplementary Figure 7. Protein levels of glia and neuron markers in the proteomic data set.** Protein abundance of glial and neuronal markers in all the samples used for proteomics analysis. Abundance is expressed in Log2 scale. Hexb: β-hexosaminidase subunit β; Fth1: Ferritin heavy chain; Pros1: Protein S; Sirpa: Signal-regulatory protein alpha-1; Gfap: Glial fibrillary acidic protein; Camk2b: Calcium/calmodulin-dependent protein kinase type II subunit beta; Dlg1: Synapse-associated protein 97 (SAP97); Mapt: Microtubule-associated protein tau; Map2: Microtubule-associated protein 2; Ppp1r9b: Neurabin-II, Protein phosphatase 1 regulatory subunit 9B or Spinophilin; Tubb3: Tubulin beta-3 chain or neuron-specific class III beta-tubulin.

**SUPPLEMENTARY TABLES**

**Supplementary table 1A. Significant Gene Ontology terms for transcriptomics data after the differentiation of Saline NSCs.**

**Supplementary table 1B. Significant Gene Ontology terms for transcriptomics data after the differentiation of Poly I:C NSCs.**

**Supplementary table 2A. Significant Gene Ontology terms for proteomics data after the differentiation of Saline NSCs.**

**Supplementary table 2B. Significant Gene Ontology terms for proteomics data after the differentiation of Poly I:C NSCs.**

**Supplementary table 3. Phosphopeptides identified by LC-MS/MS when comparing Poly I:C versus Saline cells after the differentiation process.** The excel tab “Phosphoproteomic_limma” contains all the phosphopeptides identified, with its Fold Change, pvalues, qvalues, genenames and the identification code of the peptides identified in the LC-MS/MS. The acquisition information, Uniprot accession number, sequence peptides, peptides identified, scores and additional information can be found in the tab “Phosphoproteomic_Peptides”. The excel tab “GO_Phosphopeptides” contains the phosphopeptides from Figure 4D. The excel tab “Map2_Phosphopeptides” contains all the Map2 phosphopeptides identified and mentioned in figure 5A.

**Supplementary table 4. Sample details for each RAW file deposited in GEO (transcriptomic data) or PRIDE (proteomic and phosphoproteomic data).**
